# Genetic association analysis using weighted false discovery rate approach on Genetic Analysis Workshop 18 data

**DOI:** 10.1186/1753-6561-8-S1-S76

**Published:** 2014-06-17

**Authors:** Xin Qiu, Xiaowei Shen, Osvaldo Espin-Garcia, Abul Kalam Azad, Geoffrey Liu, Wei Xu

**Affiliations:** 1 Department of Biostatistics, Princess Margaret Cancer Center, 610 University Avenue, Toronto, Ontario, M5G 2M9, Canada; 2 Ontario Cancer Institute, Princess Margaret Cancer Center, 610 University Avenue, Toronto, Ontario, M5G 2M9, Canada; 3 Dalla Lana School of Public Health, University of Toronto, 155 College Street, Toronto, M5T 3M7, Canada

## Abstract

In a genome-wide association study, association between disease trait and hundreds of thousands of genetic markers are tested. Several methods have been proposed to control the false discovery rate in such high-throughput data to adjust for multiple hypotheses testing. For Genetic Analysis Workshop 18, we applied the method of false discovery rate control with *p *value weighting on family-based association tests on quantitative trait to detect association between single-nucleotide polymorphisms (SNPs) and mean arterial pressure. This method can improve statistical power by incorporating independent but relevant information about the research objective. Using the real genetic and phenotype data of chromosome 3 from Genetic Analysis Workshop 18, 1 SNP from gene CACNA2D3 was found to have significant association with mean arterial pressure.

## Background

Recent developments in technologies have made it possible to collect a large amount of data and perform thousands of statistical tests on the data. A lot of methods have been proposed to control multiple testing. The family-wise error rate control method is too stringent. The false discovery rate (FDR) method proposed by Benjamini and Hochberg [1] is more powerful but it treats all the tests equally without any adjustment. Genovese et al proposed the weighted false discovery rate (WFDR) control method [2] to obtain an FDR-adjusted *p *value by incorporating prior information about the hypotheses. For genome-wide association studies, the *p *values for multiple testing can be adjusted by the results from previous genetic linkage study with improved power [2].

In Genetic Analysis Workshop 18 (GAW18), a family-based association test study was conducted to identify single-nucleotide polymorphisms (SNPs) that have significant association with blood pressure, a major cardiovascular and heart disease risk factor. Current genome-wide association studies have identified several genes that are associated with blood pressure or hypertension [4,5]. With no access to previous linkage analysis study, we extended the WFDR control method to adjust *p *values from family-based association test by independent information from population based association. We find this method can improve statistical power by conducting a simulation study.

## Methods

### Study population

The GAW18 data set consists of 1043 individuals from 20 Mexican American pedigrees from Type 2 Diabetes Genetic Exploration by Next-generation sequencing in Ethnic Samples (T2D-GENES) Project 2. Real genetic data for 472,049 SNPs on odd-numbered autosomes are available for 959 individuals. A total of 932 individuals have real phenotype data, including sex, age at examination, year of examination, systolic blood pressure (SBP), diastolic blood pressure (DBP), antihypertensive medications, hypertension diagnosis, and tobacco smoking for up to 4 time points. The maximal set of genetically unrelated individuals consists of 157 individuals, among which 142 individuals have real genotypes. Our study focused on real genetic data for chromosome 3 only.

### Definition of outcome

As a result of the high missing rate in real phenotype data of GAW18 for second or later examinations, all the analysis was based on phenotype information for the first examination only. For each individual ***i***, we define the quantitative phenotype as mean arterial pressure (MAP) [6], which can be determined by baseline SBP and DBP as follows:

(1) $$Y_{i}=\frac{2\times DBP_{i}+SBP_{i}}{3}$$

MAP is a term often used in medicine to describe an average blood pressure by combining DBP and SBP in an individual.

### Statistical analysis

We applied adjusted linear regression models to each of the SNPs in chromosome 3 within unrelated individuals to conduct a population-based association test. Family-based association tests for this quantitative trait were applied to all the trio families using the quantitative transmission disequilibrium test (QTDT). We then applied the WFDR control by incorporating *p *values from the population-based association test as weights to control the FDR of family-based association tests. The following material describes our analysis steps.

### Population-based association analysis

Apply the adjusted linear regression model to the unrelated subjects. The adjusted covariates are sex, age at baseline examination, use of medications, and principal component from population stratification. Thus the model fitted for each SNP is as follows:

(2) $$MAP=\alpha +\beta_{1}\times sex+\beta_{2}\times age+\beta_{3}\times medication+\beta_{4}\times PC+\gamma_{j}X_{j}$$

where $X_{j}$ represents the *j*^th ^SNP. We denote *p *value for *j*^th ^SNP as $P_{pop,j}$ and then use this information as weights to adjust *p *values from family-based association tests in the later step.

### Family-based association analysis

Break the large pedigrees into trios. A trio family consists of 2 genotyped parents and 1 offspring. The offspring should have both genotype and phenotype information. Apply family-based association tests for quantitative trait by PLINK [3] on MAP among those trio families. We denote *p *value for *j*^th ^SNP as $P_{fam,j}$ for j=1,2,…,m and adjust multiple testing according to Benjamini-Hochberg [1] method. Let $P_{\left(1\right)}<P_{\left(2\right)}<\ldots <P_{\left(m\right)}$ be ordered *p *values from m hypothesis tests, then Benjamini-Hochberg procedures rejects any null hypothesis for any P≤T with

(3) $$T=\text{max}\left\{P_{\left(j\right)}:P_{\left(j\right)}\leq \frac{\alpha j}{m}\right\}$$

### Weighted FDR approach

Apply WFDR control to adjust $P_{fam,j}$. We first assign weights $W_{j}$ proportional to $1/P_{pop,j}$ to null hypothesis for each SNP such that $\sum^{W_{j}}=m$, which in our case is

(4) $$W_{j}=\frac{m/P_{pop,j}}{\sum_{k=1}^{m}\left(1/P_{pop,k}\right)}$$

For each test ***j***, we compute $Q_{j}=P_{fam,j}\;/\;W_{j}$ and order $Q_{j}^{\prime }s$ to get $Q_{\left(1\right)}<Q_{\left(2\right)}<\ldots <Q_{\left(m\right)}$. Then we apply the Benjamini-Hochberg procedure in equation (2) at level *α *to each $Q_{\left(j\right)}$ and thus obtain adjusted *p *values.

### Power evaluation by simulations

To evaluate the performance of the WFDR approach, simulations were implemented. First, we simulated 250 trio families with a single quantitative phenotype, 49,999 SNPs were simulated under the null hypothesis in which each SNP is independent to the phenotype; 1 SNP was simulated under the alternative hypothesis to be associated with the phenotype. The association of the phenotype and genotype was based on the linear relationship

(5) phenotype=β*genotype+α+ε

where *α *is a constant, *genotype *is an ordinal variable of the SNP genotype (0 for major/major genotype, 1 for major/minor genotype, and 2 for minor/minor genotype), *ε *is random error, which follows normal distribution, and *β *is the coefficient that reflects the effect size of relationship between genotype on phenotype. We also simulated 250 independent individuals for each SNP; each SNP was used to test population-based association.

Different effect sizes were simulated for the associated SNP. We applied 1000 replications. We also applied both the ordinary FDR approach and the WFDR approach on the simulated 250 trio families. Empirical *p *values were calculated by counting how many replications were significant after applying the ordinary FDR or WFDR approach under different effect sizes.

## Results

### Genetic quality control

Among all family members, 58% of are females and the median MAP is 88, ranging from 58 to 154. Among unrelated individuals, 58% are females and the median MAP of all unrelated individuals is 89 (70 to 126).

For genetic data, we started quality control with 65,460 SNPs of chromosome 3 using PLINK [3] in both 959 family members and 142 unrelated individuals. We removed 4641 SNPs with a missing rate greater than 0.05 and 11,414 SNPs with a minor allele frequency smaller than 0.05. This left us with 49,407 SNPs and 959 family members. We further applied quality control among unrelated subjects. Remaining in the sample were 42,727 SNPs of 132 individuals, as 9 individuals with a high missing rate were excluded and 1 individual was deleted as an outlier by comparing to HapMap data. Principal components analysis [7] was used to adjust for the population stratification effect among unrelated individuals. After merging SNPs from family based and population based samples, we end up with 40,359 SNPs. Then we broke the family into trio families, resulting in 260 eligible trio families for the QTDT.

### Statistical results

**Figure 1:**
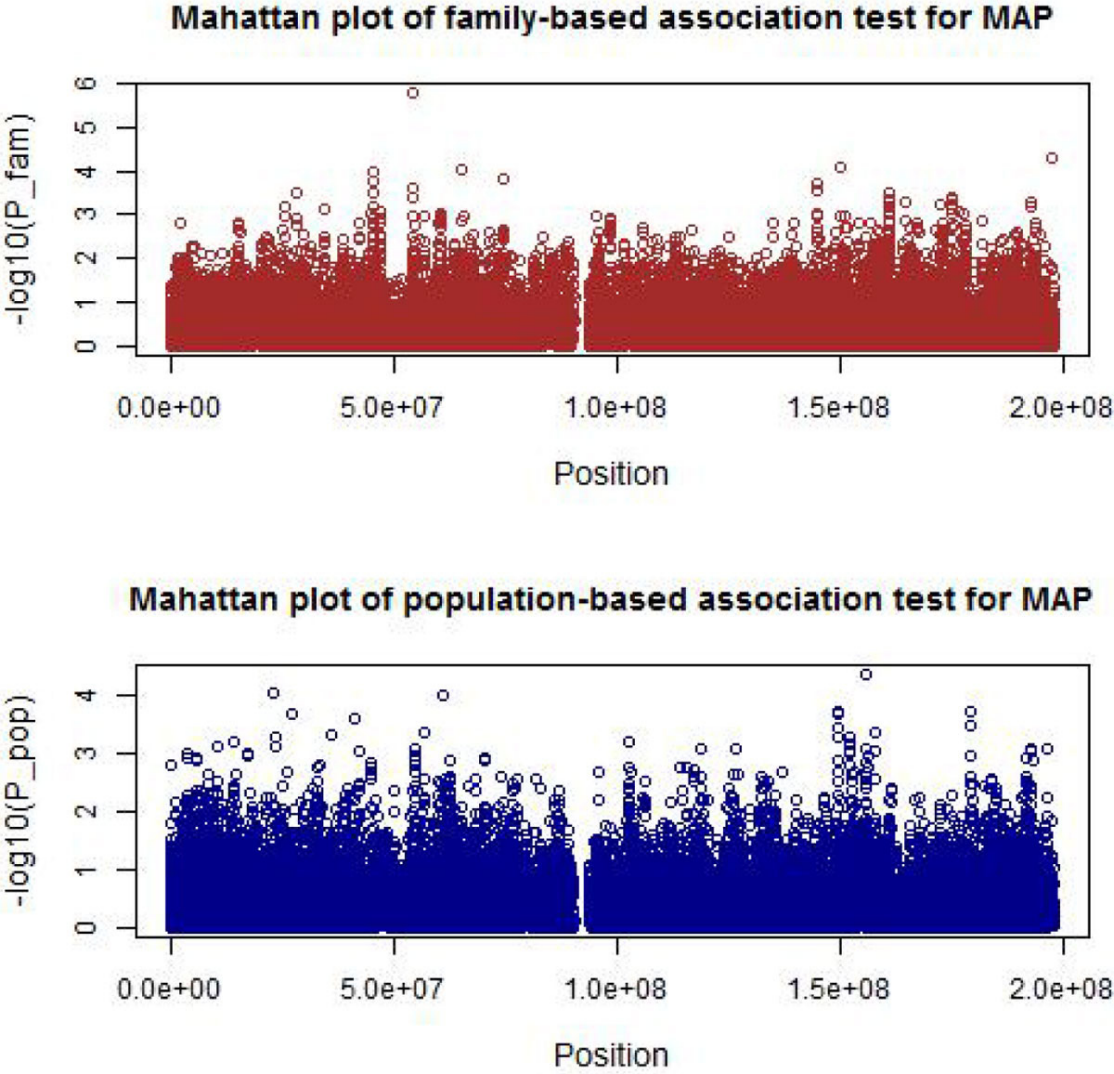
**Manhattan plot of family-based and population-based association tests for MAP**.

Our results for both family-based and population-based association tests for MAP were obtained by PLINK. *p *Values of each SNP and corresponding positions were provided in the Manhattan plots (Figure 1). The genetic model applied on each SNP was the additive model.

The population-based association analysis yielded no significant signal at an adjusted significance level of 0.05/40359 = 1.24 × 10^−6 ^after clinical factors are adjusted. Table 1 summarizes the results for the 4 top SNPs.

**Table 1:** Top 4 SNPs from population-based association analysis

Chr	SNP	Gene	BP	Minor allele	MAF (%)	*p *Value
3	rs9828558	*NA*	23115291	G	33	9.14E-05
3	rs7616789	*NA*	27024158	T	26	2.05E-04
3	rs2700464	*ULK4*	41522811	T	19	2.53E-04
3	rs12634258	*NA*	61291738	T	38	9.87E-05

BP, base pair; MAF, minor allele frequency.

The family-based association analysis identified no significant SNPs. The smallest *p *value among all SNPs is 1.59 × 10^−6^, which is larger than the adjusted significance level of 1.24 × 10^−6^. Table 2 summarizes the details of the top 4 SNPs from family-based association analyses.

**Table 2:** Top 4 SNPs from family-based association analysis

Chr	SNP	Gene	BP	Minor allele	MAF (%)	*p *Value
3	rs17638423	*LARS2*	45466958	T	14	1.04E-04
3	rs9828485	*CACNA2D3*	54288475	A	19	1.59E-06
3	rs6650908	*NA*	65148368	G	33	9.30E-05
3	rs500857	*CNTN3*	74327008	G	42	1.52E-04

BP, base pair; MAF, minor allele frequency.

After applying FDR control on the weighted *p *values from the population-based association analysis, 1 SNP, rs9828485, achieves significant level with an adjusted *p *value of 0.0312. However, there is no significant SNP if we apply ordinary FDR control (Benjamini-Hochberg) to the raw *p *values from the family-based association test. Table 3 provides more results.

**Table 3:** Comparison of different *p *values for SNP rs9828485

SNP	Gene	BP	Minor allele	MAF (%)	P_fam_	P_pop_	P_FDR_	P_WFDR_
rs9828485	*CACNA2D3*	54288475	A	19	1.59E-06	0.05	0.0624	0.0321

BP, base pair; MAF, minor allele frequency; P_fam_, p value for family-based test; P_pop_, p value for population-based test; P_FDR_, p value false discovery rate; P_WFDR_, p value for weighted false discovery rate.

### Simulation results

**Figure 2:**
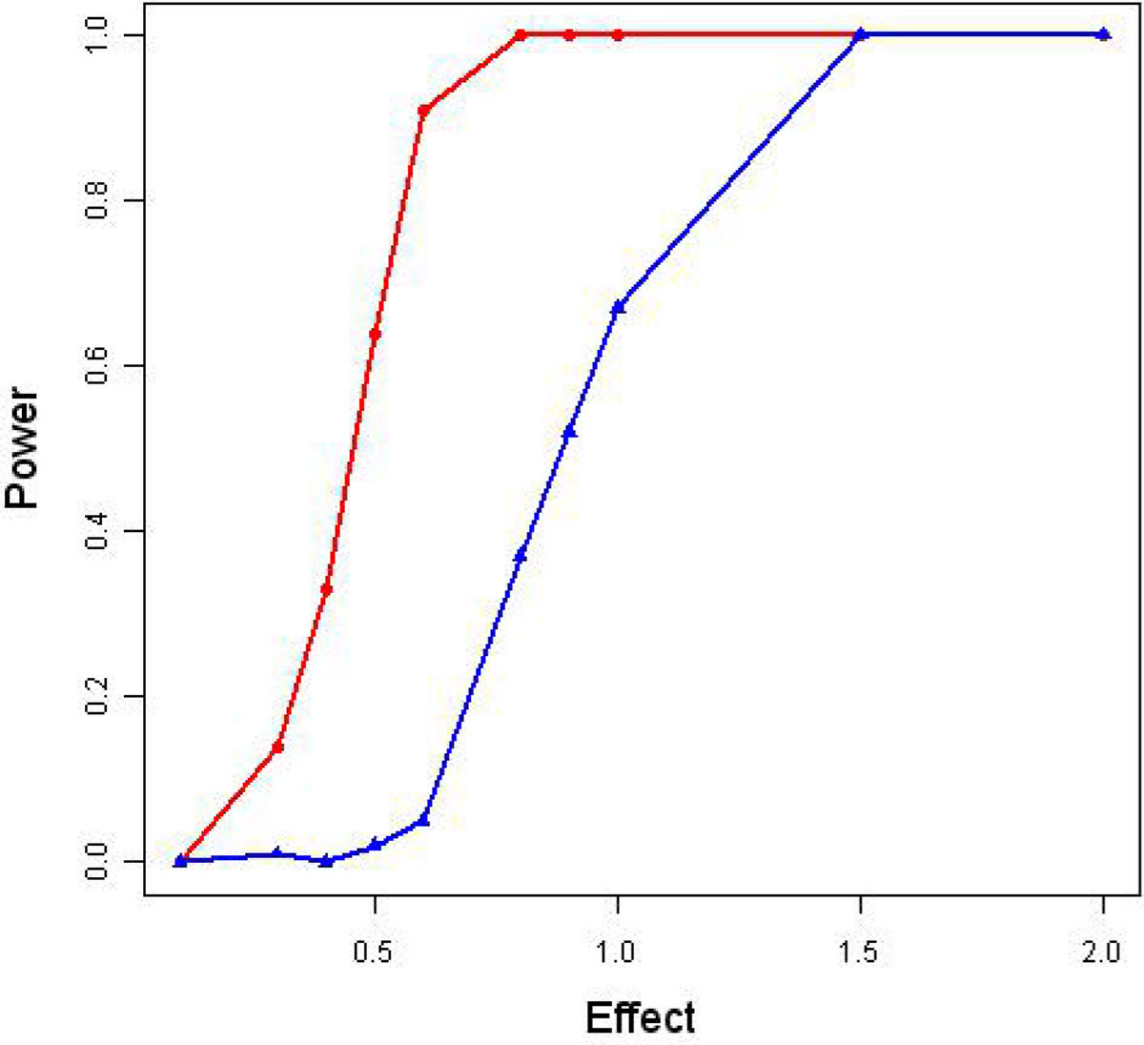
**Statistical power under different effect sizes by using ordinary FDR and WFDR**. The blue line represents the power by using FDR and the red line is for WFDR.

We applied simulation with 10 different effect sizes of *β *to assess the power under different effect sizes. We chose *β*s as 0.1, 0.3, 0.4, 0.5, 0.6, 0.8, 0.9, 1.0, 1.5 and 2.0. Figure 2 shows the empirical powers. It is easy to conclude that the power was improved by using the WFDR approach.

## Discussion

In this study, we extended the WFDR to adjust *p *values from the family-based association test by incorporating the population-based association test. A simulation study was conducted to examine the improvement of statistical power of WFDR comparing FDR under different effect sizes.

According to the simulation results, we believe that the weighted method for FDR control improves statistical power if there is a correct assignment of weights as we can see a big increase in the statistical power from FDR to WFDR when the effect size between phenotype and genotype is moderate. Although our study only focused on continuous outcome, the method can also be applied to other disease outcomes, such as binary outcome. Further analysis and simulation could be explored to assess the efficiency of the WFDR method for binary outcome. We are further developing our method for family-based association tests on the nuclear family.

## Conclusions

We propose a WFDR method to adjust *p *values for family-based association test, which we believe we will more powerful than FDR. Based on GAW18 data, we evaluated the genetic association between each single SNP from chromosome 3 and MAP using the family-based association test for quantitative trait. Using ordinary FDR to control for multiple testing, we found no significant association. However, applying WFDR, we found 1 SNP rs9828485 in gene *CACNA2D3 *that is strongly associated with MAP. *CACNA2D3 *has been reported to be associated with cardiac disease [8]. Because high blood pressure is a major risk factor for cardiac disease, *CACNA2D3 *could also lead to blood pressure.

## Competing interests

The authors declare that they have no competing interests.

## Authors' contributions

XQ and WX designed the analysis plan. XQ carried out the analysis and drafted the manuscript. XS generated genetic quality control. All authors participated in discussions of the analysis plan and helped revise the manuscript. All authors read and approved the final manuscript.
